# Germline mutations of KIT in gastrointestinal stromal tumor (GIST) and mastocytosis

**DOI:** 10.1186/s13578-016-0120-8

**Published:** 2016-10-18

**Authors:** Hengning Ke, Julhash U. Kazi, Hui Zhao, Jianmin Sun

**Affiliations:** 1 Department of Pathogen Biology and Immunology, School of Basic Medical Sciences, Ningxia Medical University, No. 1160 Shengli Street, Yinchuan, 750004 People’s Republic of China; 2 Translational Cancer Lab, General Hospital of Ningxia Medical University, Yinchuan, People’s Republic of China; 3 Division of Translational Cancer Research, Lund Stem Cell Center, Department of Laboratory Medicine, Lund University, Lund, Sweden; 4 Faculty of Medicine, School of Biomedical Sciences, The Chinese University of Hong Kong, Sha Tin, Hong Kong, People’s Republic of China

**Keywords:** KIT, Cancer, Signal transduction, Targeted therapy

## Abstract

Somatic mutations of KIT are frequently found in mastocytosis and gastrointestinal stromal tumor (GIST), while germline mutations of KIT are rare, and only found in few cases of familial GIST and mastocytosis. Although ligand-independent activation is the common feature of KIT mutations, the phenotypes mediated by various germline KIT mutations are different. Germline KIT mutations affect different tissues such as interstitial cells of Cajal (ICC), mast cells or melanocytes, and thereby lead to GIST, mastocytosis, or abnormal pigmentation. In this review, we summarize germline KIT mutations in familial mastocytosis and GIST and discuss the possible cellular context dependent transforming activity of KIT mutations.

## Background

Great progress has been made in the targeted therapy of cancer in recent years. Numerous targeted therapies have been approved for the treatment of various cancers. The number of new drugs targeting specific proteins or pathways is increasing rapidly. In many cancers, protein kinases are deregulated, and therefore, are the most often used therapeutic targets in the treatment of cancer. Gain-of-function mutations, overexpression, genomic rearrangements and autocrine activation of kinases are the frequent causes of cell transformation in most malignancies [1–3].

KIT is a receptor tyrosine kinase that is implicated in gastrointestinal stromal tumor (GIST), mastocytosis and core binding factor (CBF) acute myeloid leukemia (AML) [4]. Imatinib is a small molecule inhibitor that was originally developed to inhibit BCR-ABL fusion protein which later found to inhibit the activity of KIT [5]. Thus, imatinib was approved for the treatment of GIST [6], where it improved the treatment outcome dramatically. Due to the resistance of some primary or secondary KIT mutations to Imatinib, new inhibitors of KIT were developed. Recently, Sunitinib and Regorafenib were approved as second and third line treatment of GIST respectively [7, 8].

Mutations of KIT are the dominant genetic lesion in GIST and mastocytosis. Both somatic and germline mutations of KIT have been described in GIST, mastocytosis and other cancers [9–12]. Mutations of KIT were found in almost each domain of KIT while the distribution of the mutations is not random. There are some hotspots of somatic mutations of KIT and the hotspots in GIST and mastocytosis are different with the reason unknown.

KIT is important for the development of interstitial cells of Cajal (ICC), mast cells and melanocytes [13, 14]. However, germline mutations of KIT do not necessarily induce the transformation or overgrowth of all three types of cells and show different phenotypes in the patients, which might reflect the tissue-specific transforming ability of KIT mutations and explain the difference in the hotspots of somatic KIT mutations in different malignancies. These mutations could be good models to study tissue-specific transforming mechanism of KIT mutations and contribute to design effective targeted therapy of malignancies carrying KIT mutations. In this review, we summarize germline KIT mutations in familial mastocytosis and GIST and discuss how different KIT mutations induce cell transformation in different tissues.

## Signal transduction of wild-type KIT

KIT was cloned in 1987 as the human homolog of its viral counterpart, v-kit. The KIT gene is localized to the human chromosome 4 and on mouse chromosome 5 [15]. KIT is a member of type III receptor tyrosine kinase together with FLT3, PDGFR and CSF-1R. This family of kinases is characterized by an extracellular ligand-binding domain consisting of five immunoglobulin-like regions, a transmembrane domain, a juxtamembrane domain and an intracellular kinase domain which is separated by a short kinase insert. KIT plays important roles in melanogenesis, gametogenesis, and hematopoiesis [4]. The ligand for KIT, stem cell factor (SCF), is encoded by *sl* locus of the mouse and it was cloned in 1990 [16, 17].

Stimulation of KIT with its ligand, SCF, leads to the dimerization of receptors and activation of the intrinsic tyrosine kinase activity followed by phosphorylation of specific tyrosine residues in the intracellular domain. In KIT, several tyrosine residues including Tyr 568, Tyr 570, Tyr 703, Tyr 721, Tyr 730, Tyr 823, Tyr 900, Tyr 936 can be phosphorylated upon SCF stimulation [18–23]. Phosphorylated tyrosines, together with adjacent amino acid residues, form specific binding sites for downstream signaling molecules and activate specific downstream signaling pathways.

Phosphorylation of Tyr 568 plays a critical role in the activation of KIT and downstream signaling pathways. Phosphorylated Tyr 568 can activate Src family kinases [18, 24] and the Src family kinases in turn further enhance the activation of KIT [25]. Inhibition of Src family kinases leads to attenuation of KIT activation, indicating that the activity of Src family kinases is necessary for the complete activation of KIT. In addition, Src family kinases can activate SHC and Ras-Raf-Mek-Erk signaling cascade that is important for KIT-mediated cell proliferation [18].

Another important KIT phosphorylation site is Tyr 721, which acts as the docking site for the regulatory subunit p85 of the PI3 kinase [20]. Activation of PI3 kinase and its downstream signaling pathways regulates the KIT-mediated cell survival and proliferation [26]. The Tyr 721 is not the only site involved in PI3 kinase activation by KIT. It has been demonstrated that Tyr 703 and Tyr 936 are the binding sites for the adaptor protein Grb2, which in turn recruit PI3 kinase through Gab2 and activates downstream signaling cascades. Gab2 is also involved in activation of Ras-Raf-Mek-Erk signaling cascade [27].

Activation of KIT is tightly controlled to avoid excessive activation of downstream signaling pathways. One mechanism of negative regulation of KIT activity is Cbl-mediated ubiquitination. Cbl is an E3 ubiquitin ligase which associates with the phospho-Tyr 568 and Tyr 936 residues in KIT [28] and induces the receptor degradation and thereby attenuates the signal transduction of the receptor. In addition, Grb2 is also involved in the recruitment of Cbl to the receptor [29]. Loss of Cbl function might prolong activation of KIT and its downstream signaling pathways.

**Fig. 1:**
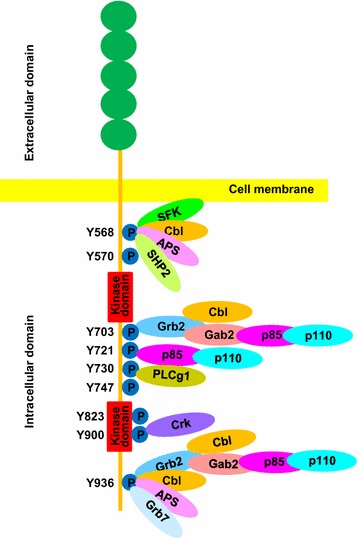
Schematic diagram of the tyrosine phosphorylation sites in wild-type c-Kit and their interaction molecules. KIT is a transmembrane receptor tyrosine kinase with an extracellular ligand-binding domain consisting of five immunoglobulin-like regions, a transmembrane domain, a juxtamembrane domain and an intracellular kinase domain which is separated by a short kinase insert. Upon binding of its ligand stem cell factor, some tyrosine sites as indicated in the intracellular domain of KIT are phosphorylated, leading to the activation of downstream signaling pathways

Phosphorylation of other KIT tyrosine residues activates specific downstream signaling pathways and contributes to KIT-mediated cell response as well. Tyr 570 enhances the binding of Src family kinases to KIT although Tyr 570 does not bind to Src family kinases directly. Tyr 730 and Tyr 900 are docking sites for PLC-gamma and Crk respectively [22, 23] that can further activate their downstream signaling pathways. The tyrosine phosphorylation of wild-type KIT and activation of downstream signaling pathways are summarized in Fig. 1.

## Signal transduction of KIT mutants

Somatic and germline mutations of KIT have been found in various malignancies which are mainly characterized by ligand-independent activation. Ligand-independent constitutive activation is considered as a cause of cell transformation induced by KIT mutants. However, recent studies suggest that KIT mutants gain extra activity in addition to the constitutive activation [30], and the activation mechanism, as well as downstream signaling pathways, are different compared to that of wild-type KIT [31–34].

The D816V mutation is the most often occurred and widely studied oncogenic KIT mutation. In addition to the ligand-independent activation, this mutation gains extra activity and it has different signaling pathways from that of wild-type KIT. For example, while the wild-type KIT activation and downstream signaling are partially dependent on Src family kinases, KIT/D816V gains Src-like kinase activity and it circumvents a requirement of Src family kinases in its signaling [30]. Similar to KIT/D816V, the exon 11 mutation V560D of KIT can be fully activated without Src family kinases although it does not have Src-like kinase activity [34]. These studies strongly suggest that oncogenic KIT mutations gain extra activity that wild-type receptor does not hold. These mutations can not only induce ligand-independent activation of the receptor but also might have different downstream signaling pathways compared with wild-type KIT. Elucidation of the activation mechanism and downstream signaling pathways of KIT mutants will contribute to drug design and targeted therapy of malignancies carrying KIT mutants.

PI3 kinase is an important downstream signaling molecule of KIT. It plays an important role in wild-type KIT-mediated cell proliferation [26], migration [35], and KIT/D816V mediated cell transformation [36]. Further study of PI3 kinase in the signal transduction of KIT mutants indicates that PI3 kinase not only activates Akt and related downstream signaling pathways, but also it plays a central role in the ligand-independent activation of KIT mutants. Blockage of the direct binding of PI3 kinase to KIT dramatically inhibits the ligand-independent activation of KIT/D816V and abolishes the transforming ability of KIT/D816V [32]. More strikingly, blockage of the direct binding of PI3 kinase to KIT completely blocks the ligand-independent activation of KIT/V560D [34], which further strengthens the key role of PI3 kinase in the ligand-independent activation of KIT mutants. Furthermore, the activity of PI3 kinase in the ligand-independent activation of KIT mutants does not rely on the lipid kinase activity of PI3 kinase [32, 34]. These data indicate that PI3 kinase can be an alternative drug target in malignancies induced by KIT mutants.

In addition to the different roles of Src family kinases and PI3 kinases in the activation of wild-type KIT and KIT mutants, the unique downstream signaling pathways of KIT mutants were studied. It has been shown that KIT/D816V, but not wild-type KIT, can induce tyrosine phosphorylation of p110delta and SLAP, and the phosphorylation of the two molecules contribute to KIT/D816V mediated cell transformation [32, 33]. The knowledge about the activation and signal transduction of KIT mutants is still very limited so far; more studies are needed to further understand the difference in the activation and downstream signaling pathways between wild-type KIT and KIT mutants.

## Somatic mutations of KIT

Mastocytosis is characterized by abnormal proliferation and accumulation of mast cells in tissues. It is divided into systemic mastocytosis and cutaneous mastocytosis according to the infiltrated tissues. Mutations of KIT account for around 80 % of mastocytosis [37–39], and can be found in almost each region of KIT but are not randomly distributed. Exon 17 mutation, D816V of KIT is the most often occurred KIT mutation in mastocytosis [37]. In addition to D816V mutation, less common oncogenic mutations including D816F, D816H, D816Y, D820G in exon 17 [40–43], exon 10 and exon 11 mutations, F522C and V559I respectively are also identified in mastocytosis [44, 45]. In CBF AML, within many KIT mutations, D816V is the dominant mutation [46].

GIST is considered originate from ICC in the digestive tract. The average diagnostic age is the mid 60s [47]. KIT mutations are also the most common mutations in GIST similar as that in mastocytosis. Unlike mastocytosis in which exon 17 mutation D816V is the dominant KIT mutation, exon 11 mutation in KIT is most common in GIST, and exon 9 and 13 mutations are also often seen in GIST but to a less extent [48, 49]. Exon 17 mutation of KIT is mainly found as a secondary mutation in drug-resistant GIST after the failure of targeted therapy [50–52].

The reason for the difference in hotspots of KIT mutations between mastocytosis and GIST remains unknown, the study of KIT mutations in different host cells might give some clues. In a previous study, it was shown that the V560D mutation of KIT cannot support the survival of hematopoietic cells in the absence of the ligand, while expression of KIT/D816V in the same cell line is enough to support the cell survival in the absence of the ligand [34]. It is worth to mention that the V560D mutation is common in GIST and the D816V mutation happens frequently in mastocytosis, a hematological malignancy [32, 34]. It is possible that hematopoietic cells are not the right host cells for the transforming activity of KIT/V560D. In contrast to its high transforming activity in hematopoietic cells, expression of KIT/D816V in fibroblast cells display weak oncogenic potential [53] and so far no D816V mutation has been reported in GIST. Therefore, it is most likely that oncogenic potential of different KIT mutants profoundly dependent on host cell type or cancer type.

## Germline mutations of KIT

**Fig. 2:**
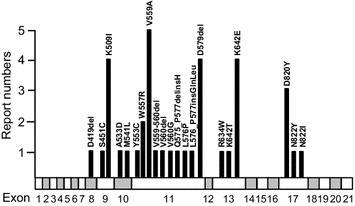
Distribution of germline KIT mutations in exons of the KIT locus. Report numbers are the numbers that the mutation was reported in familial GIST and mastocytosis

In contrast to somatic mutations, germline mutations of KIT were only found in few cases of familial mastocytosis and GIST, suggesting the transforming activity of germline mutations of KIT is limited to mast cells and ICC. So far, only 37 reports described 21 well-sequenced germline mutations in KIT (Table 1; Fig. 2).

**Table 1:** List of germline KIT mutations in familial GIST and mastocytosis

Exon	Mutation	GIST	Mastocytosis	Pigmentation	References
8	D419del	Yes	Yes	Normal	Hartmann et al. [54]
9	S451C	No	Yes	Hyperpigmentation	Wang et al. [55]
9	K509I	No	Yes	Normal	Zhang et al. [56]
9	K509I	Yes	Yes	Normal	Speight et al. [57]
9	K509I	No	Yes	Normal	de Melo Campos et al. [58]
9	K509I	No	Yes	Normal	Chan et al. [59]
10	A533D	No	Yes	Normal	Tang et al. [12]
10	M541L	No	Yes	Normal	Foster et al. [60]
11	Y553C	Yes	No	Normal	Nakai et al. [61]
11	W557R	Yes	No	Some patients have hyperpigmentation	Robson et al. [62]
11	W557R	Yes	No	Normal	Hirota et al. [63]
11	V559A	Yes	No	Hyperpigmentation	Maeyama et al. [11]
11	V559A	Yes	One patient has urticaria pigmentosa	Hyperpigmented spots	Beghini et al. [64]
11	V559A	Yes	No	Hyperpigmentation	Kuroda et al. [65]
11	V559A	Yes	One patient has urticaria pigmentosa	Lentigines, malignant melanoma	Li et al. [66]
11	V559A	Yes	No	Normal	Kim et al. [67]
11	V559-560del	Yes	No	Hyperpigmentation	Nishida et al. [68]
11	V560del	Yes	No	Normal	Bamba et al. [69]
11	V560G	Yes	No	Normal	Kang et al. [70]
11	Q575_P577delinsH	Yes	No	Normal	Wozniak et al. [71]
11	L576P	Yes	No	Hyperpigmentation	Neuhann et al. [72]
11	L576_P577insGlnLeu	Yes	No	Hyperpigmentation	Carballo et al. [73]
11	D579del	Yes	No	Normal	Jones et al. [74]
11	D579del	Yes	No	Normal	Tarn et al. [75]
11	D579del	Yes	No	Normal	Lasota et al. [76]
11	D579del	Yes	No	One patient has Hyperpigmentation	Kleinbaum et al. [77]
13	R634 W	No	Yes	Normal	Pollard et al. [78]
13	K642T	Yes	No	Normal	Yamanoi et al. [79]
13	K642E	Yes	No	One patient has nevi, lentigine	Bachet et al. [80]
13	K642E	Yes	No	Normal	Isozaki et al. [81]
13	K642E	Yes	No	Normal	Graham et al. [82]
13	K642E	Yes	No	Some patients have hyperpigmentation; some patients have paradoxical cutaneous depigmentation	Vilain et al. [83]
17	D820Y	Yes	No	Normal	Hirota et al. [84]
17	D820Y	Yes	No	Normal	Veiga et al. [85]
17	D820Y	Yes	No	Normal	O’Riain et al. [86]
17	N822Y	Yes	No	Normal	Thalheimer et al. [87]
17	N822I	No	Yes	Normal	Wasag et al. [88]

## Germline mutations of KIT in mastocytosis

Seven different germline KIT mutations in familial mastocytosis have been reported so far. In contrast to somatic KIT mutations in mastocytosis that were mainly found in exon 17, germline KIT mutations are located in exon 8, 9, 10, 13 and 17.

KIT is expressed in hematopoietic stem cells and progenitor cells and it plays important roles in the regulation of hematopoiesis. Differentiated hematopoietic cells lose expression of KIT with the exception of mast cells [89]. Interestingly, mast cells are the only hematopoietic cells that can be transformed by germline mutations of KIT. There are no any reports about deficiency in other hematopoietic cells in patients that carry germline mutations of KIT (Table 1), indicating that the transforming ability of germline mutations of KIT in hematopoietic system is limited to mast cells but not all KIT-expressing hematopoietic stem cells and progenitor cells. Sporadic mastocytosis associated leukemia has been reported [90], which indicate that somatic D816V mutation of KIT can transform other hematopoietic cells in addition to mast cells. Compared with D816V mutation, the germline mutations of KIT in familial mastocytosis are weak mutations in the hematopoietic system.

Gene mutations in sporadic mastocytosis are well studied, and KIT mutations are the major mutations of mastocytosis [91], however, the downstream signaling pathways of KIT mutants mediating transformation of mast cells remains unknown. Identification of the key signaling pathway in the transformation of mast cells will provide us novel drug targets and will contribute in developing of targeted therapy of mastocytosis. As we can see in Table 1, some germline KIT mutations, such as S451C, A533D, M541L, R634W and N822I, can induce only mastocytosis but not any other symptom that is related with a KIT mutation such as GIST, suggesting that these mutations can activate the necessary signaling pathways for mast-cell transformation. Study of these germline KIT mutation might elucidate the necessary signaling pathway that KIT mutations transform mast cells.

## Germline mutations of KIT in GIST

The gastrointestinal tract is the most affected tissue in patients carrying germline KIT mutation, as indicated in Table 1, totally 15 different germline KIT mutations have been reported in GIST. Similar to somatic mutations of KIT in GIST, germline KIT mutations in exon 11 are the most common [49]. Germline KIT mutations of Val 559 and Lys 642 are hotspots in familial GIST (Fig. 2). Both mutations were also found as somatic mutations in sporadic GIST [92, 93].

Compared with mastocytosis, targeted therapy was well developed against KIT mutations in GIST. Imatinib, Sunitinib and Regorafenib are used as first, second and third line treatment of GIST, they have dramatically improved the treatment outcome [94]. However, some primary mutations and secondary mutations of KIT are resistant to the three approved KIT inhibitors; it is necessary to further study the activation mechanism and downstream signaling pathways of KIT mutants in GIST in order to improve the treatment. Some germline mutations of KIT, such as Y553C, W557R, D579del and K642E, only induce GIST but not mastocytosis (Table 1), the study of these KIT mutants might elucidate the specific transformation mechanism of KIT mutations in ICC.

## Germline mutations of KIT in melanocyte

Melanocytes are another type of cells that are dependent on KIT for their lineage commitment, migration, and survival [95, 96]. Mutations in KIT or its ligand SCF lead to a defect in pigmentation [97]. Although BRAF mutation is the dominant mutation in melanoma, mutations in KIT gene have also been reported [98].

Only one germline mutation of KIT, V559A, has been described in melanoma so far [66], other patients in the same family carrying the same germline mutation of KIT did not develop melanoma. It is difficult to conclude that germline KIT mutation can induce melanoma based on the only observation. However, hyperpigmentation was reported in 10 cases of familial mastocytosis or GIST, suggesting that germline mutations of KIT can at least enhance the pigmentation (Table 1).

## The same germline mutation of KIT induces different diseases

Germline mutations of KIT induce either GIST or mastocytosis except that D419del can induce both [54], suggesting that these mutations might have different activation mechanism and downstream signaling pathways and that their transforming ability might be cell type dependent or cellular context dependent.

Asn 822 in exon 17 can only induce GIST but not mastocytosis when it is mutated to Tyr [87] as germline mutation, while it can only induce mastocytosis (urticaria pigmentosa) but not GIST when it is mutated into Ile [88]. It is interesting that one amino acid residue mutated into different amino acids leads to different phenotypes in the patients, which indicates possible different activation and/or downstream signaling pathways of the two different mutations in one site.

It is worth to note that some patients carrying the same germline KIT mutation, such as V559A, have different phenotypes. All family members carrying germline V559A mutation developed GIST and hyperpigmentation, meaning that V559A mutation of KIT is an oncogenic mutation in GIST and it can enhance pigmentation. Besides GIST, some patients also developed other symptoms such as urticaria pigmentosa [64, 66], and one patient even developed malignant melanoma and angioleiomyoma [66]. But these symptoms are not common among all the patients, meaning that V559A mutation of KIT cannot induce these symptoms by itself. It is possible that the mutation makes the patients sensitive to KIT mutation related other symptoms and additional factors are needed to cooperate with V559A mutation of KIT in the onset of other symptoms.

Same as V559A mutation, all patients carrying germline K642E mutation of KIT developed GIST, but they have opposite phenotypes concerning pigmentation. Some patients have nevi and lentigine [80], some patients have no abnormal pigmentation [81, 82] and other patients have paradoxical cutaneous depigmentation [83]. These different phenotypes in pigmentation suggest that K642E mutation of KIT might enhance, inhibit or have no effect on melanocyte depends on the situation. Maybe the same as V559A mutation, the phenotype might depend on other factors as well. It is interesting to identify the factor that can decide the outcome of the mutation in pigmentation. Identification of these factors will give clues about the oncogenesis of melanoma, and contribute to the treatment of melanoma.

Both V559A and K642E mutations of KIT were also identified as somatic mutations in melanoma [99, 100]. Since the patients that carry germline V559A and K642E mutations of KIT do not necessarily develop melanoma although some of them have hyperpigmentation, it can be concluded that these two mutations are not driver mutations in melanoma.

Germline K509I mutation of KIT was reported in few cases of familial mastocytosis. The patients carrying germline KIT/K509I have normal pigmentation, suggesting that the mutation probably behave similarly as wild-type KIT in melanocytes and it has on transforming activity in melanocytes. GIST is only reported in one patient but not all patients are carrying germline K509I mutation of KIT, meaning that K509I mutation of KIT is not an oncogenic mutation in GIST.

## Knockin mice carrying germline mutations of KIT

Mice are widely used animal models in life sciences. Mutations of KIT were introduced into the murine genome to study their transforming potentials. Germline D818Y mutation of murine KIT (identical to D820Y of human KIT) was made in mice. Mice carrying both homozygous and heterozygous D818Y mutation of KIT developed GIST [101], they recapitulated the phenotype showed by patients carrying germline D820Y mutation of KIT. No disorder in mast cells and pigmentation was reported in the knockin mice; that is in line with the phenotype of the patients carrying germline D820Y mutation of human KIT.

Deletion of V558 in KIT was also generated in murine genome. Mice carrying heterozygous V558del mutation of KIT developed GIST [102]. In addition, these mice also had increased the amount of mast cells in tissues although they did not develop mastocytosis. Which means that this mutation can enhance the proliferation of mast cells although it cannot transform mast cells, the transforming activity of V558del mutation of KIT is limited in ICC.

Similar as above two mutations, knockin mice harboring a K641E mutation of KIT (identical to K642E mutation of human KIT) also developed GIST [103], indicating that K642E mutation of KIT is a driver mutation in GIST. Interestingly, mice carrying a homozygous K642E mutation of KIT showed loss-of-function phenotypes in pigmentation, hematopoiesis and gametogenesis. These mice had white fur, very few mast cells and they were infertile. The loss-of-function phenotypes strongly suggest that K624E mutation of KIT is a loss-of-function mutation in melanocytes, hematopoietic cells and germ cells. It is interesting that one mutation can act as both gain-of-function mutation and loss-of-function mutation. The mechanism behind that might reflect the tissue-specific activation mechanism of KIT mutations and further explain the difference in hotspots of KIT mutations between GIST and mastocytosis. The germline mutations of KIT carried by the patients is usually heterozygous, maybe one copy of wild-type KIT is enough to support normal pigmentation, hematopoiesis and gametogenesis as showed by mice carrying a heterozygous K641E mutation of KIT. From the different phenotypes showed by knockin mice carrying heterozygous and homozygous K641E mutation of KIT, we can know that patients carrying germline mutations of KIT cannot always precisely reflect the function of KIT mutations since the mutations carried by these patients are usually heterozygous. Homozygous knockin mice are sometimes necessary to confirm the role of KIT mutations.

## Conclusions

The different phenotypes mediated by various germline mutations of KIT might reflect the cellular context dependent transforming ability of kit mutations and explain the difference in hotspots of KIT mutations between GIST and mastocytosis. Germline mutations of KIT can give the information about the necessary signaling pathways in the transformation of a certain type of cells. Study on the activation and downstream signaling pathways of germline KIT mutations will elucidate the tissue-specific transformation mechanism of KIT mutations, and which will further contribute to the developing of targeted therapy of malignancies that carry KIT mutations.
